# Ionotropic Crustacean Olfactory Receptors

**DOI:** 10.1371/journal.pone.0060551

**Published:** 2013-04-03

**Authors:** Elizabeth A. Corey, Yuriy Bobkov, Kirill Ukhanov, Barry W. Ache

**Affiliations:** 1 Whitney Laboratory, Center for Smell and Taste, and McKnight Brain Institute, University of Florida, Gainesville, Florida, United States of America; 2 Departments of Biology and Neuroscience, University of Florida, Gainesville, Florida, United States of America; Duke University, United States of America

## Abstract

The nature of the olfactory receptor in crustaceans, a major group of arthropods, has remained elusive. We report that spiny lobsters, *Panulirus argus*, express ionotropic receptors (IRs), the insect chemosensory variants of ionotropic glutamate receptors. Unlike insects IRs, which are expressed in a specific subset of olfactory cells, two lobster IR subunits are expressed in most, if not all, lobster olfactory receptor neurons (ORNs), as confirmed by antibody labeling and *in situ* hybridization. Ligand-specific ORN responses visualized by calcium imaging are consistent with a restricted expression pattern found for other potential subunits, suggesting that cell-specific expression of uncommon IR subunits determines the ligand sensitivity of individual cells. IRs are the only type of olfactory receptor that we have detected in spiny lobster olfactory tissue, suggesting that they likely mediate olfactory signaling. Given long-standing evidence for G protein-mediated signaling in activation of lobster ORNs, this finding raises the interesting specter that IRs act in concert with second messenger-mediated signaling.

## Introduction

Our understanding of the fundamental basis of olfactory coding and signal processing was revolutionized by the discovery of the nature of the olfactory receptor, initially in mammals [1] and subsequently in numerous species of animals (Reviews: [2], [3]). While crustaceans, a major group of arthropods, have been useful physiological models for studying olfaction, the nature of their olfactory receptors has remained elusive. Early evidence that olfactory signal transduction in lobsters involved G protein-mediated second messenger signaling [4], [5], [6], [7] triggered homology searches against mammalian olfactory G protein-coupled receptors, but to no avail. Searches for orthologs of the traditional insect olfactory receptors/coreceptor (Ors/Orco) subunits, odorant binding proteins, and gustatory receptors (GRs) also were without success.

However, early differential analyses of mRNAs from the lobster olfactory organ revealed fragments that were similar to traditional ionotropic glutamate receptors (iGluRs) such as kainate, N-methyl-D-aspartate and AMPA receptors [8], [9]. These fragments were suspected to represent potential modulatory receptors on the soma of lobster ORNs for glutamate since modulatory ionotropic gamma-aminobutyric acid (GABA) [10] and histamine [11] receptors were the focus of ongoing research at the time. The recent finding that some insect olfactory receptors, known as IRs, are similar in structure to iGluRs [12] offered the interesting possibility that these crustacean iGluRs may actually function as olfactory receptors in crustaceans. The prospect that crustacean olfactory receptors are orthologs of insect IRs received further support from the fact that the *Daphnia* genome revealed abundant IRs [13], but no traditional Ors/Orco [14].

Here we show that two IR subunit genes, *PargIR25a* and *PargIR93a*, are expressed in most or all spiny lobster (*Panulirus argus*) ORNs, as confirmed by *in situ* hybridization. PargIR25a, can be localized to the transduction compartment (outer dendrites) of the ORNs by western blot and immunocytochemistry. Restricted ligand-specific responses visualized by calcium imaging are consistent with the limited expression of the non-IR25a/IR93a subunits, suggesting that cell-specific expression of the uncommon IR subunits determines the ligand sensitivity of a given cell. These results argue for IR-mediated olfactory signaling in lobster ORNs and that IRs mediate a primary, if not the sole, odorant input to these cells. The lobster model will allow us to study these important receptors, which can be expected to mediate important behaviors such as host-seeking in insects and larval fouling in marine crustaceans, and how they are involved in olfactory transduction.

## Results

### Identification of Multiple IRs, but no Ors/Orco or GRs, in Lobster ORNs

It has been previously suggested that the lobster iGluR1 amino acid sequence bears a strong similarity to that of the broadly expressed *Drosophila* IR subunit IR25a [13]. Based on the previously sequenced American lobster (*Homarus americanu*s) iGluR1 and iGluR2 genes [8], [15], we used homology-based cloning to obtain two full length spiny lobster iGluR gene sequences from a cDNA library and compared them to both the American lobster iGluR and *Drosophila* IR sequences (Table 1). The spiny lobster iGluR1 sequence is 78.6% similar at the nucleotide level and 82.0% similar at the predicted amino acid level to the American lobster sequence. Based on its similarity of 51.5% at the amino acid level to *Drosophila* IR25a (Figure S1), we have named the spiny lobster sequence *PargIR25a*. The spiny lobster iGluR2 subunit has 39.4% similarity to *Drosophila* IR8a and 79.3% identity with the American lobster iGluR2 subunit at the amino acid level and has been named *PargIR8a*. Sequence alignment with predicted IR sequences from *Daphnia pulex* results in 51.3% similarity for IR25a and no predicted IRs of significant similarity for IR8a. The online program tblastn (National Center for Biotechnology Information, Bethesda, MD; http://www.ncbi.nlm.nih.gov.lp.hscl.ufl.edu/BLAST/) was used to search for ESTs encoding putative IRs in other crustaceans using the spiny lobster IR25a and IR8a sequences. The program database was set to non-human, non-mouse ESTs (EST_others) and restricted to crustacean transcripts (taxid: 6657). All hits were checked manually for homology to the target query. Blast sequence similarity searching with PargIR25a and PargIR8a predicted amino acid sequences indicates that there are IR-like sequences in additional crustaceans such as *Calanus finmarchicus*, *Petrolisthes cinctipes*, *Lepeophtheirus salmonis*, *Callinectes sapidus*, and *Armadillidium vulgare*.

**Table 1:** Comparison of predicted amino acid sequences of lobster and *Drosophila* IRs.

	PargIR25a	PargIR8a	PargIR93a
**DmelIR25a**	51.5%	24%	15.4%
**DmelIR8a**	24.2%	39.4%	14.1%
**DmelIR93a**	13%	11.8%	23.9%
**PargIR25a**	100%	24.7%	15.7%
**PargIR8a**	24.7%	100%	14.4%
**PargIR93a**	15.7%	14.4%	100%
**HameIR25a**	82%	25.2%	15%
**HameIR8a**	25.9%	79.3%	15%

*Drosophila* (Dmel), *Panulirus argus* (Parg) and *Homarus americanus* (Hame) IR predicted amino acid sequences were compared. Geneious software, version 5.6.6 created by Biomatters (available from http://www.geneious.com/) was used to translate putative protein sequences and align sequences (using the Geneious ClustalW plug-in) to generate percent amino acid identity. Accession number include KC595306 for PargIR25a, KC595307 for PargIR8a, KC595308 for PargIR93a, AY098942.1 for HameIR25a, NM_135019.2 for DmelIR25a, NM_167185.1 for DmelIR8a, and NM_142666.4 for DmelIR93a. The HameIR8a sequence is unpublished (generously shared by Dr. Timothy McClintock).

**Figure 1:**
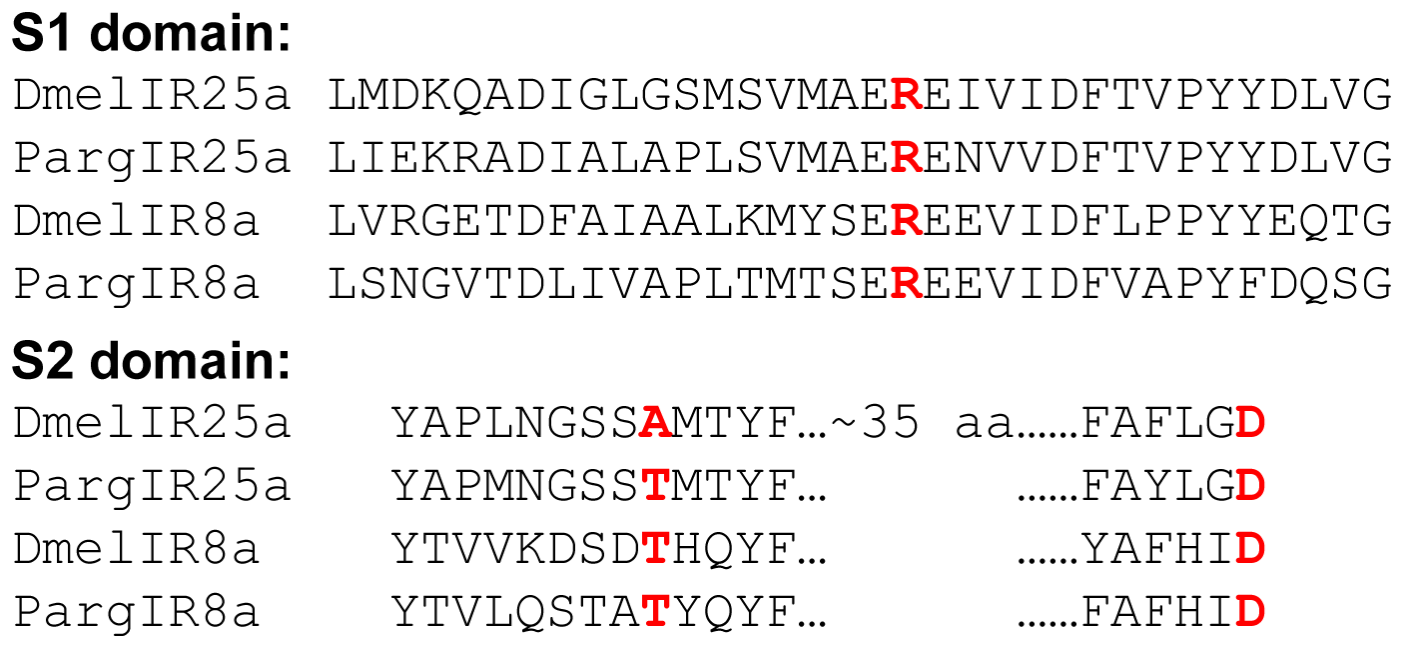
Alignment of spiny lobster IR25a and IR8a S1 and S2 ligand binding domains. Spiny lobster and *Drosophila* S1 and S2 ligand binding domains were manually aligned. Putative ligand binding residues are highlighted in red and bolded.

Structural analysis and alignment of the spiny lobster IR25a and IR8a predicted amino acid sequences reveals that they have a predicted protein structure similar to that of iGluRs and insect IRs with an extracellular two-lobed ligand-binding domain, three transmembrane regions, an ion channel pore and a cytoplasmic C-terminal domain. When considering just the S1 and S2 ligand binding domains of the IRs, the arginine (R), threonine, and aspartate/glutamate characteristic glutamate binding residues are completely conserved in only 3 of the 61 predicted protein sequences from *Drosophila*, one of which is IR8a. Two of the three residues are conserved in *Drosophila* IR25a. Similar to the *Drosophila* IR8a sequence, all three residues are conserved in the predicted amino acid sequences of spiny lobster IR25a and IR8a (Figure 1).

Based on our identification of the lobster IR25a and IR8a subunits as possible olfactory receptors, a spiny lobster olfactory cDNA library was subjected to high-throughput 454 sequencing to try to identify additional potential olfactory receptor subunits. We focused in particular on identifying receptor genes despite the diversity of the sequences available. BLAST sequence similarity searching of the olfactory transcriptome with protein sequences from the highly conserved and ubiquitous Orco of insect olfactory receptors failed to identify any similar sequences. Similar results were obtained with searches with insect ligand-specific Or subunits, GRs and odorant binding proteins, as well as with vertebrate olfactory receptors and trace amine-associated receptors. In contrast, we identified hundreds of partial sequences that align with the insect IR family and lobster iGluR sequences, ranging from 22 to 100% identity at the amino acid level to the spiny lobster IR25a sequence. Of these, the majority were identical to the nucleotide sequence of *PargIR25a*. Despite its broad pattern of expression in *Drosophila* ORNs and identification in lobster olfactory cDNA, no transcripts of *PargIR8a* were detected in the olfactory transcriptome, indicating that while it may be present in the olfactory tissue, it is expressed either at a low level or in a limited number of cells.

Within the IR sequences in the transcriptome database, an additional 42 S1 ligand binding domains can be identified (Figure S2). Although in *Drosophila* only 19 IRs (31%) retain the R residue in S1 that interacts with glutamate in iGluRs, this residue is conserved in approximately 80% of the lobster IR S1 sequences. Of the lobster S2 sequences that we have obtained, the glutamate binding residues are not conserved other than in PargIR25a and PargIR8a. As the S2 domain has a much more variable sequence, we have only tentatively identified these sequences in the transcriptome and better understanding of conservation of the glutamate binding residues in this domain will have to await full length cloning and sequencing of the subunits. Although some of the S1 ligand binding domains identified may be from traditional iGluRs, we would predict that many are from chemosensory IRs. The variability of the ligand binding domain sequences and lack of conservation between the insect and crustacean sequences likely reflects differences in odorant specificity.

Using RACE, we have obtained longer sequences of additional IR subunits, including *PargIR93a*, *PargIR4*, and *PargIR7.* The predicted protein sequences of the full length lobster IRs are most closely related to other lobster IR sequences, with 37.9% amino acid identity between PargIR4 and PargIR7, but all have a predicted structure similar to traditional iGluRs. Although its strongest level of similarity is with *Drosophila* IR93a, PargIR93a is only 23.9% similar to *Drosophila* subunit at the amino acid level. The remaining sequences are not sufficiently similar to any named insect IR sequences to allow identification of their homologous subunits and have been named based on the order of their identification in lobster. Although the *D. pulex* genome has been sequenced [16], no genomes are currently available for marine crustaceans. Given the low level of nucleotide sequence conservation between *D. pulex* and lobster IRs, as well as the difficulty of obtaining full length sequences by RACE, additional subunits are still in the process of being fully sequenced and cloned with the goal of heterologous expression.

### IRs are Expressed in Olfactory Tissue

**Figure 2:**
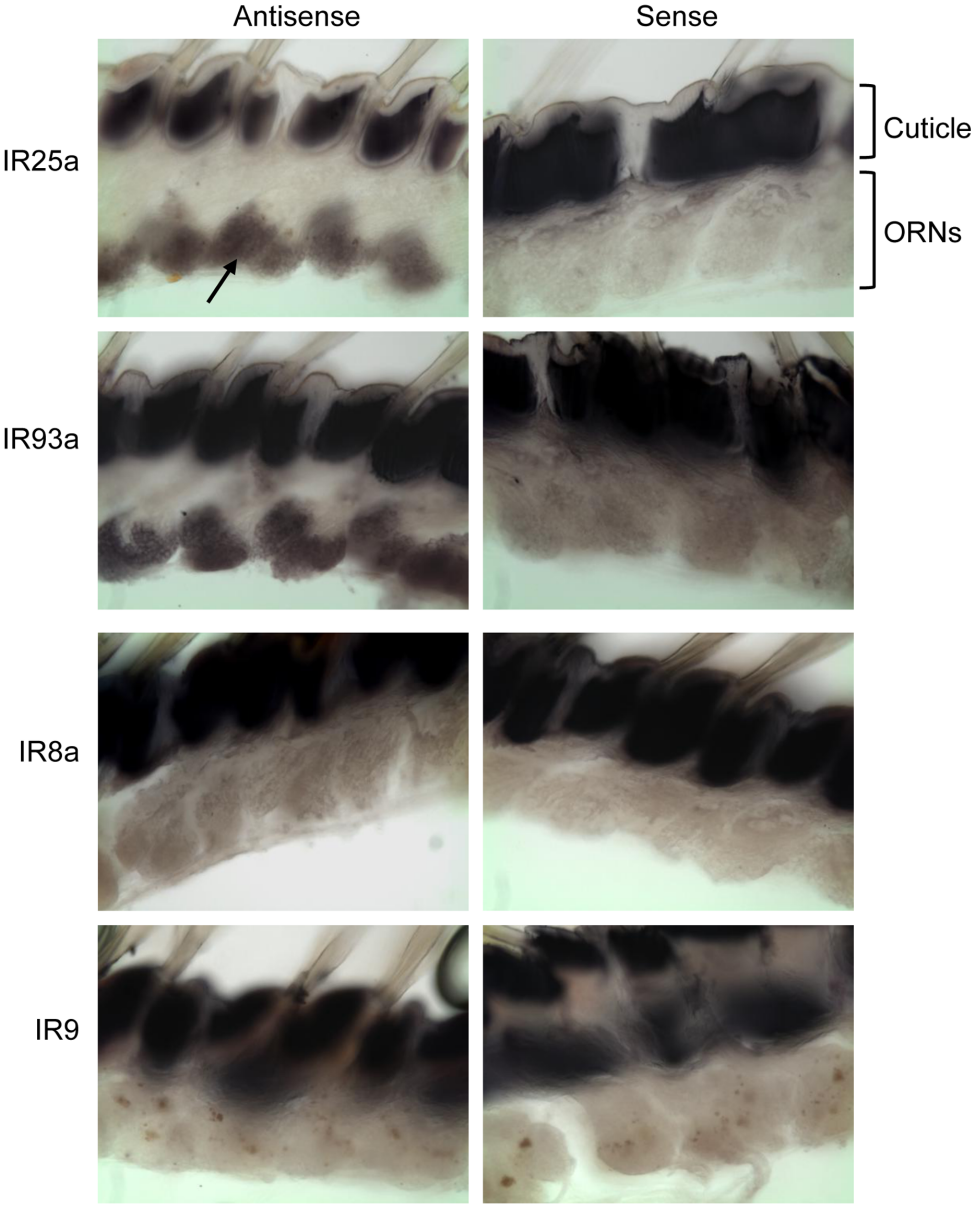
Spiny lobster IR25a and IR93a transcripts can be localized to the ORNs. *In situ* hybridization of vibratome sections of gelatin embedded lobster olfactory tissue. Antisense probes for *PargIR25a* and *PargIR93a* label most, if not all, mature ORNs. In contrast, no labeling was detectable with probes for other IRs. Specifically labeled cell bodies are indicated with an arrow in the first panel. Representative sections labeled with antisense probes for *PargIR8a* and *PargIR9* are shown. No specific labeling was detected with the sense probes for the same gene regions. The cuticle in each section is non-specifically labeled.

**Figure 3:**
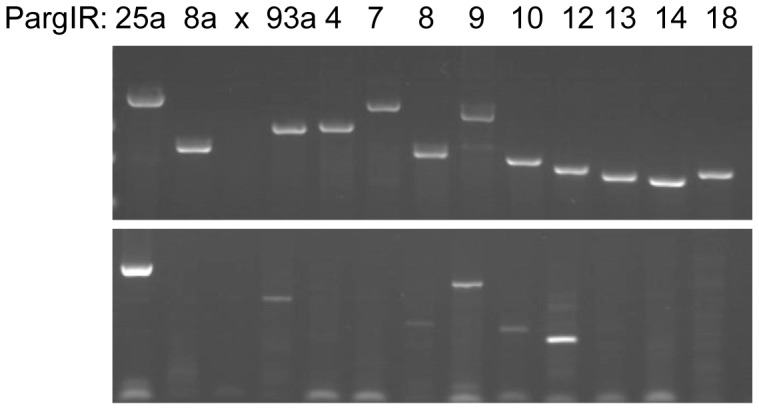
Spiny lobster IR transcripts can be detected in single ORN clusters. RT-PCR detection of IRs in RNA prepared from (top panel) total olfactory tissue and (bottom panel) a single ORN cluster. While all of the IRs tested could be detected in the total RNA sample, only a limited number could be detected in the single cluster. No amplification was detected in RNA samples in the absence of reverse transcription or template (not shown). Numbers above panels indicate the IR amplified in the wells below. X indicates empty well.

*In situ* hybridization was used to localize expression of the spiny lobster IR genes within the olfactory tissue (Figure 2). While as previously shown [17], *PargIR25a* appears to be expressed specifically in all ORNs. *PargIR93a*, the only other subunit detected by *in situ* hybridization shows a similar pattern of localization, indicating that in a majority of ORNs, IR25a and IR93a are co-expressed. An additional 8 IR gene transcripts, including *PargIR8a*, were undetectable by this method suggesting that either they are not truly present or that their expression is constrained to either a limited number of cells or a low abundance of mRNA. Given that many of the transcripts were undetectable by *in situ* hybridization, we confirmed that the IRs detected in the transcriptome but not found by *in situ* hybridization were present in the olfactory tissue using RT-PCR to test for mRNA expression in ORN clusters dissected from lobster olfactory tissue (Figure 3, top panel). All of the IRs tested could be detected by this method in samples including cDNA prepared from the entire olfactory organ, but none of the gene fragments were amplified from RNA in the absence of RT or template. Some of the IRs, though detectable in the total olfactory sample, could not be detected in cDNA prepared from individual clusters or small numbers of ORNs (Figure 3, bottom panel). These results suggest that the transcripts for these IRs are present in the olfactory tissue, but at a level that is not detectable by *in situ* hybridization, either resulting from a low level overall expression levels or an extremely restricted expression pattern. Both *PargIR25a* and *PargIR93a* could be amplified from individual ORNs, supporting their co-localization to most, and likely all, of the cells of this type (data not shown).

### IR25a is Expressed in the Transduction Compartment (Outer Dendrites) of the ORNs

**Figure 4:**
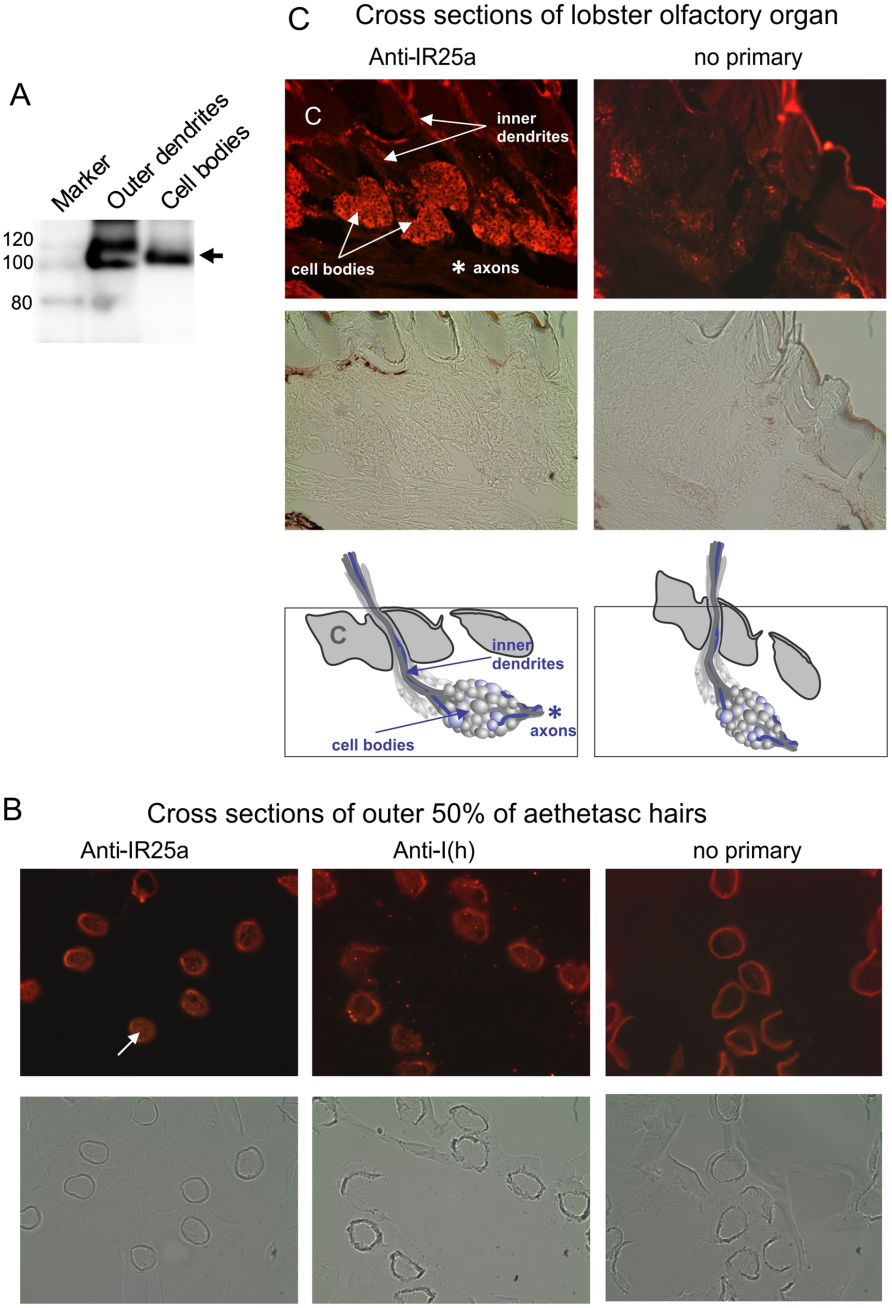
Spiny lobster IR25a can be immunolocalized by the transduction compartment (outer dendrites) of ORNs. (A) Western blot detection of IR25a in the proteins from the outer dendrites. Detergent lysates were prepared from the outer 50% of aesthetasc hairs after manual removal of the guard hairs from the lobster olfactory organ and ORN cell bodies collected from the same region. PargIR25a was detected with an anti-iGluR1 (anti-IR25a) antibody (generously provided by Dr. Timothy McClintock) after SDS-PAGE and transfer of proteins to a nitrocellulose membrane. The IR25a protein band is indicated with an arrow. (B) Immunolocalization of IR25a to the outer dendrite tissue within sections of the aesthetasc hairs of the lobster olfactory organ. Autofluorescent cuticle surrounds the outer dendrite tissue (white arrow). Controls included labeling with an anti-I(h) channel antibody and no primary antibody. Cryosections were prepared from the outer 50% of the aethetasc hairs. (C) Immunolocalization to the cell bodies and inner dendrites. In the first panel, cell bodies and inner dendrites of ORNs are indicated with arrows. Axons are indicated with an asterisk. The cuticle is indicated with a white C. The bottom two panels are diagrams showing the orientation of the sections directly above them. Cryosections were prepared from 8 annuli segments of the olfactory organ. Immunolocalization in both the outer dendrites and tissue cross sections was performed with the same antibody used in (A).

Immunolocalization of lobster iGluR1 detected it in the cell bodies and axons of the ORNs [17], and a later study also localized it to the inner dendrites [18]. Given that this expression pattern is not necessarily consistent with what is expected of olfactory receptors, we used the antibody (anti-iGluR1; anti-IR25a) made against the American lobster iGluR1 protein [17] to more closely investigate expression in the outer dendrites (transduction compartment) of the spiny lobster. Immunoreactivity of the antibody was found by western blot in proteins from the distal 50% of the aesthetasc sensilla known to contain only the outer dendrites of the ORNs (Figure 4A). Consistent with previous studies [17], the protein was also detected in protein from the cell bodies. It is interesting to note that the molecular weight of the spiny lobster IR25a protein (100 kDa) is consistent with that of one of the proteins identified as part of putative taurine receptor proteins found in dendritic plasma membrane using radioligand-receptor crosslinking assays [19].

Localization of the PargIR25a protein to the outer dendrites was further confirmed by immunocytochemistry. Immunoreactivity with the IR25a antibody occurred in cross-sections made through the distal 50% of the aesthetasc sensilla (Figure 4B). Anti-IR25a labeling was detected in the outer dendrite tissue within the autofluorescent cuticles and no IR25a labeling is apparent in the absence of the primary antibody. No IR25a labeling was detected in sections in which there was no tissue remaining within the cuticle. This pattern of labeling is consistent with that of other proteins previously localized to the outer dendrites, such as the I(h) channel (Figure 4B) [7], [20], [21]. Cross sections of the entire olfactory organ (lateral filament of the antennule) showed a pattern of distribution consistent with the results of the western blot with strong labeling of the inner dendrites and cell bodies, but not the axons as previously shown [17], [18] (Figure 4C).

### IRs are Expressed in Other Known Chemosensory, but not Non-chemosensory, Tissues

**Figure 5:**
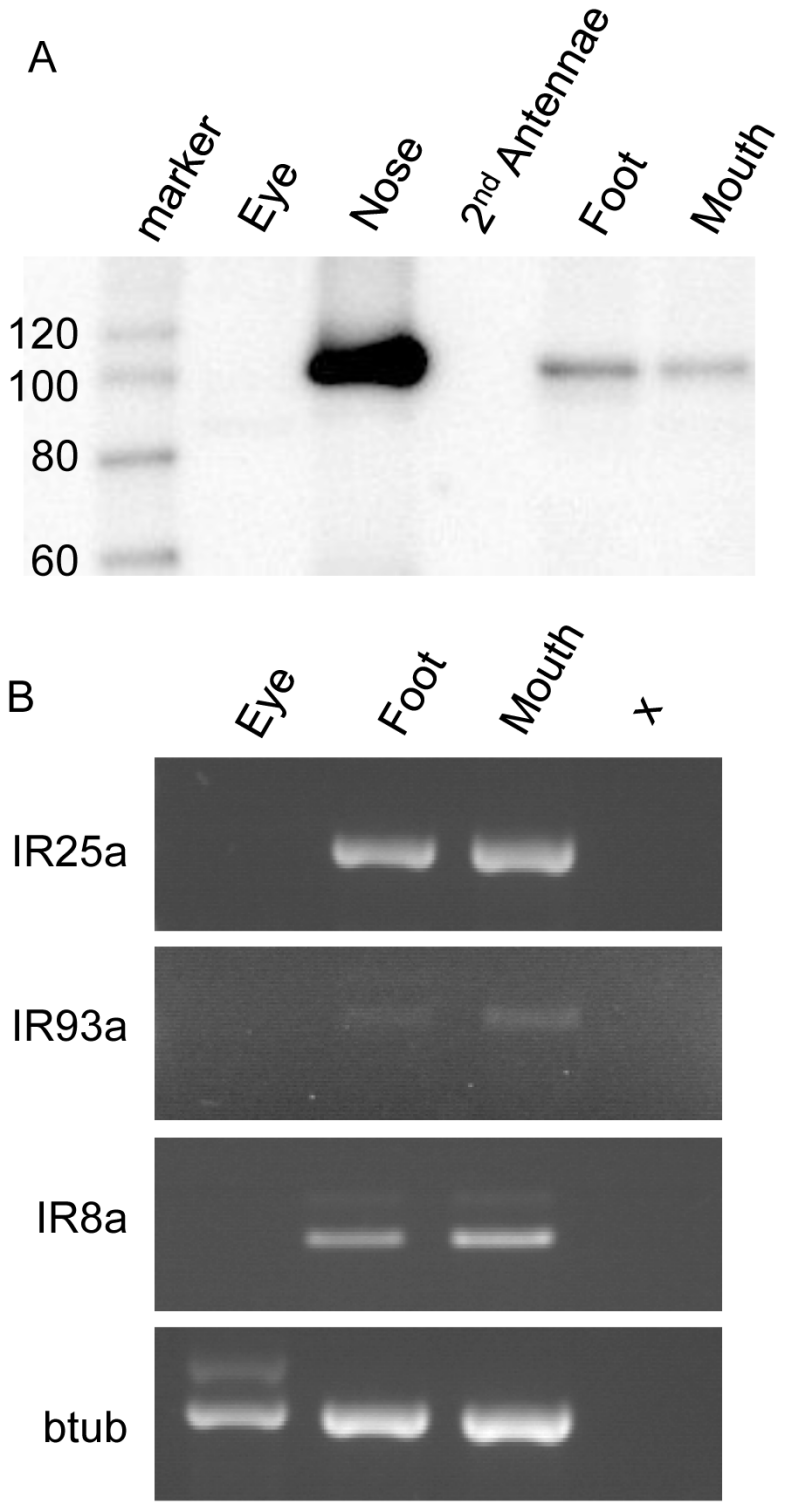
Lobster IRs can be detected in non-olfactory chemosensory tissues. (A) Western blot detection of PargIR25a in detergent lysates of *Panulirus argus* olfactory, foot and mouth tissues. No expression could be detected in lysates from the eye or non-olfactory second antenna. (B) RT-PCR detection of *PargIR25a*, *PargIR93a, PargIR8a*, and *beta-tubulin* (btub) gene expression in *Panulirus argus* eye, foot and mouth tissues. No amplification was detected in RNA samples in the absence of reverse transcription (data not shown) or template (X).

American lobster iGluR1 was localized solely to the olfactory tissue [17]. In contrast, in the spiny lobster using western blot we also found the protein to be present in other chemosensory tissues, including the mouth and walking feet (Figure 5A), potentially suggesting a common mechanism of chemosensory signal transduction. Consistent with the previous finding, PargIR25a protein was not detectable in non-chemosensory tissues such as the second antennae and eye. We further tested for IR expression in non-olfactory tissue by RT-PCR. Consistent with the western blots, the *PargIR25a* transcript could be detected in other chemosensory tissues (Figure 5B), and as would be expected if IRs serve as chemosensory receptors in other tissues, expression of the other IRs is also not restricted to the ORNs. Both commonly expressed *PargIR25a* and *PargIR93a* and olfactory-restricted subunits, such as *IR8a*, could be detected in other tissues using this method.

### Single Odorants Activate Only a Restricted Number of Lobster ORNs in Situ

**Figure 6:**
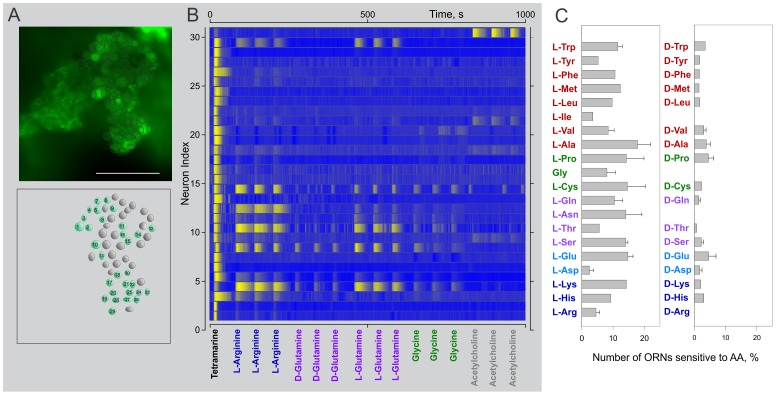
Single odorants specifically activate a restricted number of lobster ORNs. ORN ensemble activity from single neuronal cluster (A) evoked by both complex odor mixture and single odorants (B). A – Map of individual cell regions analyzed. Position of every ORN region was carefully selected and corrected, if necessary, during the recording course to maximally avoid overlapping of optical signals. B – Fluorescence intensity traces from indexed ORN regions (A, bottom) were color coded using the intensity range characterizing individual ORN. Color gradient code applied to all data points changes from blue (minimum value) to yellow (maximum fluorescent intensity value). Each prospective odor, except TET, was applied 3 times. Stimulus pulse duration was 1s in all cases. Time between successive sweeps, 60 sec. Delay between successive trials, ∼120 s (not shown). Note, while complex odor mixture would activate majority of ORNs (first sweep), single odorants evoke calcium responses in a restricted number of ORNs. In some cases, ORNs are predominantly sensitive to single odorants (e.g. ORN30 demonstrates robust responses exclusively to acetylcholine). All stimuli were used at concentration 1 mM except an aqueous extract of TET (∼0.2 mg/ml). C – Incidence histogram of the effects of L- (left column) and D- amino acid isomers (right column). Bars represent a number of ORNs sensitive to a particular amino acid expressed as percentage of a number of TET activated ORNs. Overall 10.3+−1% of ORNs are sensitive to L-AAs while in average only 2.3+−0.3% responded to D- isomers. Amino acids are grouped and color coded based on their side chains properties. D-Isoleucine and D-Asparagine were not tested.

Calcium signals can be recorded from lobster ORNs to characterize ensemble activity and detail heterogeneity in the responses of individual ORNs to odorants [22], [23]. We therefore used calcium imaging to explore in more detail the tuning to single odorants (Figure 6), including amino acids, acetylcholine, and GABA, based on the saliency of these compounds as natural chemosensory signals [24]. Consistent with previous studies [22], [23], the majority of the ORNs responded to a complex odorant mixture (TET – Figure 6A and B, first trial, only 30 ORNs shown, see also Movie S1). In contrast, significantly fewer cells responded to single odorants, even at high (1mM) concentration (e.g. L-arginine, ORNs 3, 4, 5, 8, 10, 12, 14, and 29 in Figure 6B, see also Movies S2, S3, S4), ranging from responding to several (e.g. ORNs 8 and 14 in Figure 6B), to a few (e.g. ORNs 3,12, 30 in Figure 6B) to none of the ligands tested (e.g. ORNs 2,6,17, Figure 6B).

To further evaluate ORN specificity to single odorants, we calculated the fraction of cells responsive to each single odorant as percentage of the number of mixture (TET) responsive cells (2265 cells from 46 clusters in total). The cells in each cluster were tested with 2 to 10 odorants applied in random order and the data pooled [25]. As with insect ORNs [26], [27], [28], lobster ORNs showed ligand-specific response patterning (e.g., ORN14, Figure 6B), but here we only considered the total number of cells that responded to a particular odorant. On average only 7.8±0.6% of the ORNs responded to individual odorants, ranging from 0% for GABA (1 mM) and carbachol (1 mM) to 17.8±4.2% and 25.8±3.1% for L-alanine (1 mM) and histamine (0.4 mM), respectively, further supporting our initial observations that single odorants activate restricted subsets of lobster ORNs.

Consistent with earlier suggestions [29], lobster ORNs differentially responded to L- and D-stereoisomers (Figure 6C). Overall 10.3±1% of the ORNs responded to L-isomers, but only 2.3±0.3% responded to D-isomers. The same selectivity was reflected in the response to 20 L- and 17 D-stereoisomers of the amino acids tested. For example, 14±0.8% of the ORNs responded to L-serine and 10.4±2.6% to L-glutamine vs 2.2±0.7% and 1.4±0.6%, respectively, for the D-isomers of the same amino acids (Figure 6C). These results underscore the specificity of lobster ORs for their ligands.

## Discussion

Localization of IR expression to the transduction compartment of the ORNs and their sequence similarity to insect IRs strongly imply that these receptors are indeed the elusive crustacean olfactory receptors. Given that IR subunits are expressed in the olfactory tissue of two divergent lobster genera, *Homarus* [8], [15] and *Panulirus*, and that a previous study identified IRs in *Daphnia* [13], IRs likely play a general role in initiating chemosensory signaling in crustaceans. Indeed, we have found that IR subunits similar to lobster IR25a can be identified in the publically accessible EST and cDNA sequences of other aquatic and terrestrial crustaceans such as shrimp, copepods and crabs.

As shown by this and other studies [22], [23], [30], [31], lobster ORNs respond to diverse ligands with a high level of specificity. While mammalian iGluRs form both homo- and heteromers [32], insect IRs are thought to function primarily as heteromeric receptors formed by two to three subunits [33]. This organization is consistent with the expression pattern of spiny lobster IRs. Our data indicate that PargIR25a and PargIR93a are common subunits of lobster IRs that would be predicted to form heteromers with one or more ORN-specific subunits. This would suggest that, as in insects [33], the odorant specificity of individual lobster ORNs is determined by the specific set of subunits that they express. The restricted ligand-specific responses visualized by calcium imaging are consistent with the limited expression pattern of the non-IR25a/IR93a subunits, suggesting that cell-specific expression of the uncommon IR subunits determines the ligand sensitivity of a given cell. It should be noted that our functional assessment of the lobster ORN specificity yields upper limit estimates due to the extremely high concentration of odorants tested. Because lobster IRs are expressed in most if not all ORNs and are the only type of olfactory receptor that can be detected in these cells, it can be reasonably assumed that the ligand specific-response patterns and olfactory signaling in the lobster are exclusively mediated through IRs. While it was surprising that no insect-like GRs, which are the predominant chemosensory receptor found in *Daphnia* [34], were detected in our lobster olfactory transcriptome, it remains that GRs may be expressed and mediate chemosensory signaling in the gustatory or other chemosensory tissues of the lobster.

There is long standing evidence that both excitatory and inhibitory olfactory signaling in lobster ORNs is mediated by G protein-activated second messenger pathways. Activation of lobster ORNs is GTP-dependent and can be blocked by antisera specific for Gαq [4]. Odorant-evoked excitatory signaling in lobster ORNs involves activation of phosphoinositide-3-kinase and phospholipase C [7], [20]. Further, activation of cyclic nucleotide signaling appears to mediate odor-evoked inhibitory signaling in these cells [35], [36]. This leads to the intriguing possibility that both ionotropic and metabotropic signaling contribute to the output of lobster ORNs and that ionotropic receptors act in concert with metabotropic signaling in this system. The idea that ionotropic olfactory receptors initiate metabotropic signaling has been proposed for traditional ionotropic insect Ors/Orco [37] and is consistent with evidence that ionotropic insect Ors/Orco can activate G proteins *in vitro* [38] and can activate PLC *in vivo* [39], [40], although it remains highly controversial [2], [41]. However, this idea is not novel for ionotropic receptors in that it has been proposed for some types of mammalian ionotropic glutamate receptors [42], [43], [44]. For example, GABA release in the supraoptic nucleus of the hypothalamus is mediated by kainate iGluRs through an ionotropic mode of action, whereas its inhibition is mediated by a phospholipase C-dependent metabotropic pathway activated by the same receptors [45]. The mechanism that allows the receptors to switch between the two modes of signaling, however, is not well understood.

*Drosophila* IRs respond primarily to specific amines and acids, including L-glutamate and L-glycine [46], indicating that the effective ligands for lobster ORNs are consistent with the known ligands for insect IRs and supporting our identification of IRs as crustacean olfactory receptors. Crustaceans such as the spiny lobster detect and respond to numerous chemical signals in their environment, including small, nitrogen-containing compounds such as amino acids, amines, nucleotides and peptides, which are abundant in their prey [24], suggesting that these are salient ligands. Divergent IRs may also play a role in insect taste detection [46], consistent with our ability to detect IR expression in not only olfactory tissues, but in other types of known chemosensory tissues and supporting the idea that IRs may play a more general role in arthropod chemosensation than just mediating detection of olfactory ligands.

Heterologous expression and deorphanization of lobster IRs will allow us to compare the odorant specificity and pharmacological sensitivity of the receptors with that of native ORNs. Interestingly, both heterologously expressed insect Ors/Orco [47], [48] and IRs [33] and the olfactory activity of lobster ORNs [49] are similarly susceptible to blockade by amiloride derivatives. Given that lobster olfactory activity is likely mediated by IRs, one can assume that lobster IRs will be similarly sensitive to amiloride derivatives. Shared pharmacological susceptibilities between Ors/Orco and IRs suggests similar parameters of Ors/Orco and IR pore forming structures or a novel functional mechanism - co-assembly with a ubiquitously expressed, conserved ion transporting system. This idea would be consistent with the recent finding that some TRP channels and ion exchangers form integral supra-molecular complexes with reciprocal pharmacology [50]. While speculative in the context of the current study, the shared sensitivity of insect Ors/Orco, insect IRs, and lobster IRs to amiloride and its analogs helps to underscore potential functional similarity among ionotropic olfactory receptors that is yet to be explored.

IR ligands are physiologically and behaviorally important to a growing number of insect species, such that IRs potentially present a new set of targets for the control of disease vector and agricultural pest insects [46]. Indeed, it has been argued that given their specificity for water-soluble hydrophilic acids and amines IRs act as a “independent olfactory subsystem” that mediates the host-seeking behavior evoked by these ligands in insects such as mosquitoes [46], [51]. This argument gains traction from evidence that IRs play a role in the olfactory system of aquatic mosquito larvae [52]. Our results potentially extend this idea to the control of larval settling by fouling marine crustaceans. Given the ability to patch-clamp lobster ORNs and study the underlying receptor currents directly, lobster ORNs offer a unique opportunity to study the detailed mechanisms by which IRs mediate olfactory transduction that can be linked to medically and economically important behaviors such as host seeking and larval settling in arthropods.

## Experimental Procedures

### Animals and Tissue Collection

Male spiny lobsters (*Panulirus argus*) were collected in the Florida Keys and kept in the laboratory in flowing seawater at 20−23°C on a diet of shrimp. All necessary permits were obtained. Lobsters were collected and retained under a Special Activity License issued by the Florida Fish and Wildlife Conservation Commission/Division of Marine Fisheries Management. Tissues for molecular and biochemical experiments were dissected from the mature zone of the lobster olfactory organ. We also collected other tissues from lobsters, including: walking feet, second antenna, eye, mouth, central nervous system, and stomatogastric ganglion.

### IR Identification

Amplified cDNA was generated from total RNA using the Marathon cDNA amplification kit (Clontech). The first strand synthesis utilized the AMV Reverse Transcriptase and an oligo(dT) primer. After second strand synthesis, the entire sample of cDNA was used for library construction for 454 sequencing according to manufacturers’ procedures. IR genes were identified by BlastN and BlastP searches of our original 454 transcriptome datasets with lobster iGluR and insect IR sequences. RT-PCR was used to confirm expression of the IRs in lobster olfactory tissue and RACE was used to clone full length genes for sequencing following standard methods. Multiple clones were sequenced for each gene and full length sequences are publically available in Genbank. Accession numbers include: KC595306 for PargIR25a, KC595307 for PargIR8a, KC595308for *PargIR93a*, KC603903 for *PargIR4*, and KC603904 for *PargIR7*. Individual and clusters of ORNs were lysed in RT-buffer and then oligo dT-primed RT was performed using a Verso cDNA kit (ThermoFisher) according to the manufacturer’s directions. PCR was performed with gene specific primers. Un-transcribed cell lysates were used as a negative PCR control. All PCR products were cloned and sequenced to confirm their identity.

### In situ Hybridization

Olfactory organs were cut into segments eight to ten annuli in length in saline (460 mM NaCl, 13 mM KCl, 13.6 mM CaCl_2_, 10 mM MgCl_2_, 14 mM Na_2_SO_4_, 3 mM HEPES, 1.7 mM glucose, pH 7.4). The segments were incubated in 4% paraformaldehyde (PFA) in 0.1M Sörenson's buffer (SPB), pH 7.4, for 1 hr followed by incubation for 5 to 7 days in 0.5M EDTA (pH 8.0) for decalcification. Twenty-four hrs before hybridization, annuli were embedded in 15% gelatin and fixed overnight in 4% PFA in PBS. Sections of 50 µm thickness were cut from each block with a vibratome. *In situ* hybridization was performed as previously described [8]. Sense and antisense RNA probes were prepared by transcription from pGEMT (Promega) cloned fragments using a digoxygenin labeling kit (Roche Molecular Biochemicals) and SP6 or T7 RNA polymerase. The sections were cover-slipped with Fluormount (Southern Biotechnology) and visualized with a 10x and an oil immersion 60x lens.

### Western Blotting

Proteins were run on polyacrylamide gels and transferred to nitrocellulose membranes. The membranes were blocked for 1 hr in 5% milk in PBS with 0.05% Tween 20 (PBS-T) and then incubated overnight with primary antibody diluted 1∶10000 in 1% milk in PBS-T at 4°C. The membranes were washed 6×10 min with PBS-T, followed by incubation with the appropriate horseradish peroxidase-conjugated secondary antibody (KPL) diluted 1∶20000 in 1% milk in PBS-T for 2 hrs. The membranes were washed again, incubated with ECL detection reagent (Millipore) and the signal captured with a Fluor-S Multi-Imager (Bio-Rad).

### Immunocytochemistry

Immunocytochemistry was performed following a modification of previously described methods [21]. Briefly, lobster olfactory organs were cut into segments eight annuli in length, fixed overnight in 4% PFA, the cuticle was softened in 0.5 M EDTA for 2 days, and then the tissue was soaked in 30% sucrose. The tissue was embedded in 15% gelatin, overlaid with 4% PFA in PBS and allowed to stand at 4°C for 90 min. The gelatin blocks were embedded in OCT embedding medium and frozen at −80°C. Four µm cryostat sections were made through the distal 50% of the aesthetasc hairs or 14 µm cryostat sections were made lengthwise through the segment. The slides were incubated for 10 min in PBS supplemented with 50 mM ammonium chloride. After blocking for 1 hr with 1% gelatin in PBS, the sections were incubated overnight with primary antibody diluted 1∶5000 in 1% gelatin in PBS and then washed in PBS. The sections were then incubated with fluorescently-labeled secondary antibodies (KPL) in 1% gelatin in PBS and then washed with PBS. The sections were mounted with Fluormount (Southern Biotechnology) and visualized with a 10x and an oil immersion 60x lens.

### Calcium Imaging

Lobster ORNs were imaged *in situ* as previously described [22], [23]. Briefly, a single annulus was excised from the lateral antennular filament and the cuticle on the side opposite from the olfactory sensilla (aesthetascs) was removed to provide better access to the cell bodies of the ORNs (Fig. 6A). After enzymatic treatment and cleaning the preparation was placed in *Panulirus* saline (PS, mM: 486 NaCl, 5 KCl, 13.6 CaCl_2_, 9.8 MgCl_2_ and 10 HEPES, pH 7.8–8.0.) containing the fluorescent calcium indicator Fluo - 4 AM (Invitrogen) at 5–15 µM prepared with 0.2–0.06% Pluronic F-127 (Invitrogen) for ∼1 hr at room temperature. The specimens were then transferred into fresh PS, mounted on a plastic-bottom 35 mm Petri dish and placed on the stage of an inverted microscope (Olympus IX-71) equipped with a cooled CCD camera (ORCA R2, Hamamatsu). ORNs were continuously superfused with PS using two gravity fed perfusion contours. The stimulating contour washing the sensilla (∼250 µl/min) was switched rapidly using a multi-channel rapid solution changer (RSC-160, Bio-Logic) under the software control of Clampex 9 (Molecular Devices). Stimulus duration was 1 sec. All stimuli were purchased from Sigma-Aldrich and used at concentration 1 mM except an aqueous extract of a commercially available marine fish food (Tetra Marine, TET, Tetra Werke, ∼0.2 mg/ml).

Fluorescence imaging was performed under the control of Imaging Workbench 6 software (INDEC Systems). Stored time series image stacks were analyzed off-line using Imaging Workbench 6, Clampfit 10.3, SigmaPlot 11 or exported as TIFF files into ImageJ 1.42 (available from public domain at http://rsbweb.nih.gov/ij/index.html). Continuous traces of multiple responses were compensated for slow drift of the baseline fluorescence. For illustration and analysis purposes the fluorescent traces were color coded using the intensity range characterizing individual ORN. Color gradient code applied to all data points changes from blue (minimum value) to yellow (maximum fluorescent intensity value). All recordings were performed at room temperature (22–25°C).

## Supporting Information

Figure S1 **Alignment of predicted amino acid sequences for spiny lobster and** ***Drosophila*** **IR25a and IR8a.** Geneious software, version 5.6.6 created by Biomatters (Available from http://www.geneious.com/) was used to trim low-quality sequence end reads, create consensus gene sequences, translate putative protein sequences, and align sequences (using the Geneious ClustalW plug-in).
(PDF)

Figure S2 **Alignment of potential lobster IR S1 binding domains.** BLAST sequence similarity searching of the olfactory transcriptome with predicted protein sequences of PargIR25a, PargIR8a, and PargIR93a, as well as from other partially sequenced lobster IRs, revealed additional potential S1 binding domains. Where possible sequences are manually aligned based on putative residue involved in ligand binding (bold/underlined).
(PDF)

Movie S1 **Representative fluorescence imaging time series recorded from a single cluster of the lobster ORNs loaded with fluo-4/AM (shown in** **Fig.6** **).** Pseudocolor images represent the relative change in fluorescence intensity corrected for the background. The image stacks were processed equally to accentuate variable magnitude of the response to different odorants. The cells were repetitively stimulated with Tetra Marine (TET, 0.2 mg/ml) applied for 1 s. Time counter displays real time of the recording whereby a short subtitle indicates the application of the stimulus.
(AVI)

Movie S2 **Representative fluorescence imaging time series recorded from a single cluster of the lobster ORNs loaded with fluo-4/AM (shown in** **Fig.6** **).** Pseudocolor images represent the relative change in fluorescence intensity corrected for the background. The image stacks were processed equally to accentuate variable magnitude of the response to different odorants. The cells were repetitively stimulated with L-arginine (1 mM) applied for 1 s. Time counter displays real time of the recording whereby a short subtitle indicates the application of the stimulus.
(AVI)

Movie S3 **Representative fluorescence imaging time series recorded from a single cluster of the lobster ORNs loaded with fluo-4/AM (shown in** **Fig.6** **).** Pseudocolor images represent the relative change in fluorescence intensity corrected for the background. The image stacks were processed equally to accentuate variable magnitude of the response to different odorants. The cells were repetitively stimulated with L-aspartate (1 mM) applied for 1 s. Time counter displays real time of the recording whereby a short subtitle indicates the application of the stimulus.
(AVI)

Movie S4 **Representative fluorescence imaging time series recorded from a single cluster of the lobster ORNs loaded with fluo-4/AM (shown in** **Fig.6** **).** Pseudocolor images represent the relative change in fluorescence intensity corrected for the background. The image stacks were processed equally to accentuate variable magnitude of the response to different odorants. The cells were repetitively stimulated with acetylcholine (ACH, 1 mM) applied for 1 s. Time counter displays real time of the recording whereby a short subtitle indicates the application of the stimulus.
(AVI)
