# Supplementary material for: Ionotropic Crustacean Olfactory Receptors
Source: PLoS One. 2013 Apr 3;8(4):e60551. doi: 10.1371/journal.pone.0060551 (PMC3615998; doi:10.1371/journal.pone.0060551)
Supplement: Figure S1 — Alignment of predicted amino acid sequences for spiny lobster and Drosophila IR25a and IR8a. Geneious software, version 5.6.6 created by Biomatters (Available from http://www.geneious.com/) was used to trim low-quality sequence end reads, create consensus gene sequences, translate putative protein sequences, and align sequences (using the Geneious ClustalW plug-in). (PDF) [file pone.0060551.s001.pdf]

```
            1         10        20        30        40        50        60
            |         |         |         |         |         |         |
DmelIR25a   -MG--SRLDWGVADVALWAIADQIDYHQVFINEVDNEPAAKAVEVVLTYLKKNIRYGLSV
PargIR25a   MVGGAQLLQFLYLTATLLVGAFAQKVNIFVLHDEGNVQAQQGIMAAIKFLENSADGVTVG
 DmelIR8a   -----MELPLLVLLLALRFAGSEVLKITFWIEPVQRAEFDTDIAMVLKELDALR------
PargIR8a    MEGTTMLAPLCCLCLLLLPAAVAKLTIVSALDESLQEGVAWLEEAVSRAQRSTN------

DmelIR25a   QLDSIEANK-SDAKVLLEAICNKYATSIEKKQTPHLILDTTKSGIASETVKSFTQALGLP
PargIR25a   RQDMMTMKKGDDIEATVNATCDMMDAAIQENEPPHIVLDATNTGMISETVKSFTKALALP
 DmelIR8a   --------------------------LDVKVDDTTLTLTRSEDGLDMQRFCEILSTVGAS
PargIR8a    --------------------------FTLRQEVVKVSIDNEEDGK--QTICSAALNKGAG

DmelIR25a   TISASYGQQGDLRQWRDLDEAKQKYLLQVMPPADIIPEAIRSIVIHMNITNAAILYDDSF
PargIR25a   TFAASYGQEGDIREWRDLTEEGEKVLVQLMPPGDIIVQAIRDIVRTQNITNAGIIYDDTF
 DmelIR8a   AVIDLT--YSHWEEGYNLVRSLGIGYVRLERIMRPFLDMFGDFMRQKRANNVAMVFMNAR
PargIR8a    LVVDVT--AGGWTYARETAADQDTPYLRVQISNYQWMAATDQLLQNRNATDAALIFASEA

DmelIR25a   VMDHKYKSLLQNIQTRHVITAIAKDGKREREEQIEKLRNLDINNFFILGTLQSIRMVLES
PargIR25a   VMEHKYKSLLQNLPCRHILDMWEAR-EMDLRKQMKRLQDADIVNYFAVGSRDTISRILDA
 DmelIR8a   DAVEAMQQMLVGYPFRTLIMDASQTDPGQHFLERIRSLRPAPTYIALFARAAAMNGIFEK
PargIR8a    ELDQALYYLVEGSVVRVIVISGIDAT----LTHTLKKMRPSPSYYVILGATDELNTLFSK

DmelIR25a   VKP-AYFERNFAWHAITQNEGEISSQRDNATIMFMKPMAYTQYRDRLGLLRTTYNLNEEP
PargIR25a   ATANDLFGRKYAWYAVSKDNEDIQCGCENASVVFLRPQPNADTRGRLNMLQRDFQLTATP
 DmelIR8a   VQKADLFQRPLEWHFVFLDTRDRVFKYRRQAELCTRFTLNP-RAICRSMPMPDLYCGSG-
PargIR8a    AVRNNLVTRDSRWTLVATDHKSDEFDRDMLTESTGVTLMTPAQEVCCTVRNRNTNCDCDT

DmelIR25a   QLSSAFYFDLALRSFLTIKEMLQSGAWPKDMEYLNCDDFQGGNTPQRNLDLRDYFTKITE
PargIR25a   EIDSAFYFDYTIRGIKAAAKMATEGKY-DNFKYVRCEEFDEQDPP-----VRENFDLRSA
 DmelIR8a   FTMQRAMLLNVLRSLINAAQVSPGYPLAIYQDCNATASSSEVSDP-----LEKDDYNWLD
PargIR8a    LMIPRELTVNAMLMLARVLKAND--QQNGGGDGGDQPSTTSFYSA-----LRTELNSWQN

DmelIR25a   PTSYGTFDLVTQSTQPFNGHSFMKFEMDINVLQIRGGSSVNSKSIGKWISGLNSELIVKD
PargIR25a   LKSVSVVDTWAPISWGGNGNTFLDIPLTMDKFMVLQGRTAERKALGEWDSGMPGTLMFKS
 DmelIR8a   MVHWSNFLAYAPPLPHIQDQFQSPVPGLTFAVNISAGYYSSEHEAKTDLAAWSSVGEMRL
PargIR8a    MKWEQDTLQMVP----------------QFMFDITA----INSTDTSAVGSWTTADKLRL

DmelIR25a   EEQMKNLTADTVYRIFTVVQAPFIMR------------DETAPKGYKGYCIDLINEIAAI
PargIR25a   GMSLVDFQAVTVYRITTVQQSPFIYKRT----------NEDGEVEYYGYCIDLINEIKEI
 DmelIR8a   LNETIS-PARRFFRIGTAESIPWSYLRREEGTGELIRDRSGLP-IWEGYCIDFIIRLSQK
PargIR8a    NPEYEHSPIRRFFKIGTIMARPWVSPIS--GSSVRKKPTSSNPDDYMGYCVELAARLAEK

DmelIR25a   VHFDYTIQEVEDGKFGNMDENGQWNGIVKKLMDKQADIGLGSMSVMAEREIVIDFTVPYY
PargIR25a   VDFEYELFEAPDGKFGTMNDDMEWNGMIKQLIDKQADIALAPLSVMAERENVVDFTVPYY
 DmelIR8a   LNFEFEIVAPEVGHMGELNELGEWDGVVGDLVRGETDFAIAALKMYSEREEVIDFLPPYY
PargIR8a    MKFDFEFKFPSDGQYGARQKNGSWNGLVGDLSNGVTDLIVAPLTMTSEREEVIDFVAPYF

DmelIR25a   DLVGITIMMQRPSSPSSLFKFLTVLETNVWLCILAAYFFTSFLMWIFDRWSPYSYQNNRE
PargIR25a   DLVGITILMKKPKVPTSLFKFLTVLEPEVWICILFAYAFTSVLLWIFDRFSPYSYQNNKE
 DmelIR8a   EQTGISIAIRKPVRRTSLFKFMTVLRLEVWLSIVAALVGTAIMIWFMDKYSPYSSRNNRQ
PargIR8a    DQSGISIAMRKRQREESLFKFMTVLKAEVWVSIVAALVVTGIMIWLLDRYSPYSAQNNAD

DmelIR25a   KYKDDEEKREFNLKECLWFCMTSLTPQGGGEAPKNLSGRLVAATWWLFGFIIIASYTANL
PargIR25a   RYKDDDEKREFTFKECLWFCMTSLTPQGGGEAPKNLSGRLVAATWWLFGFIIIASYTANL
 DmelIR8a   AYP--YACREFTLRESFWFALTSFTPQGGGEAPKAISGRMLVAAYWLFVVLMLATFTANL
PargIR8a    LYP--PHCRKFTLKESFWFALTSFTPQGGGEAPKALSGRTLVAAYWLFVVLMLATFTANL

DmelIR25a   AAFLTVSRLDTPVESLDDLAKQYKILYAPLNGSSAMTYFERMSNIEQMFYEIWKDLSLND
PargIR25a   AAFLTVSRLDTPIESLDDLSNQYKVQYAPMNGSSTMTYFERMAYIEKKFYEIWKDMSLND
 DmelIR8a   AAFLTVERMQTPVQSLEQLARQSRINYTVVKDSDTHQYFVNMKFAEDTLYRMWKELALNA
PargIR8a    AAFLTVERMQTPVSSLDELAGQSKINYTVLQSTATYQYFFNMAAAEEELYRVWKELTLNS

DmelIR25a   SLTAVERSKLAVWDYPVSDKYTKMWQAMQEAKLPATLDEAVARVRNSTAAT-GFAFLGDA
PargIR25a   SMSDVERAKLAVWDYPVSDKYTKMWQSMQEAGLPQTFESALERVRKSTSSSEGFAYLGDA
 DmelIR8a   SK---DFKKFRIWDYPIKEQYGHILLAINSSQPVADAKEGFANVDAHENAD--YAFIHDS
PargIR8a    TS---DQSKYRVWDYPVKEQYTHILQVIEESGPVDTAEVGFQRVLDNANGE--FAFIHDA

DmelIR25a   TDIRYLQLTNCDLQVVGEEFSRKPYAIAVQQGSHLKDQFNNAILTLLNKRQLEKLKEKWW
PargIR25a   TDIRYQVLTNCDLQIVGEEFSRKPYAIAVQQGSPLKDQFNDAILKLLNQRKLETLKERWW
 DmelIR8a   AEIKYEITRNCNLTEVGEVFAEQPYAVAVQQGSHLGDELSYAILELQKDRFFEELKAKYW
PargIR8a    SEIRYEVYHDCLLTEVGEPFAEQPYAIAVQQGSHLQDEISRTILELQKDRYFEALSAQFW

DmelIR25a   KNDEALAKCDKPEDQSDGISIQNIGGVFIVIFVGIGMACITLVFEYWWYRYRKNPRIIDV
PargIR25a   NQNPEKKVCEKDDDQSDGISIENIGGVFIVIFVGIGLACVTLAFEYWWYKHRKGPRVTDS
 DmelIR8a   NQS-NLPNCPLSEDQ-EGITLESLGGVFIATLFGLVLAMMTLGMEVLYYKKKQNALEITQ
PargIR8a    NSS-ARGPCPNDNDS-EGITLQSLGGVFIATLIGLALAMLALAAEVLYYRR---------

DmelIR25a   AEANAERSNAADHPGKLVDGVILGHSGEKFEKSKAALRPRFNQYPATFKPRF--------
PargIR25a   AKVLAVR----EYPTNDVDKSNFTTDKSNFNTEYTTGGDGYDIAPVANPW*---------
 DmelIR8a   VRPVNDSS---GSGGNSSTAPPTATSTTKQAWHIPVLEAEEKPAKVSPPPSFETATFRGK
PargIR8a    -------------GGTSQVTDITPKKALKMAVER------DHVARINVTPVY*-------

DmelIR25a   ------------------------------------
PargIR25a   ------------------------------------
 DmelIR8a   KLPARITLGDGKFKPRHGLYARRNLGASDSHSGYME
PargIR8a    ------------------------------------
```
