# Supplementary material for: Ionotropic Crustacean Olfactory Receptors
Source: PLoS One. 2013 Apr 3;8(4):e60551. doi: 10.1371/journal.pone.0060551 (PMC3615998; doi:10.1371/journal.pone.0060551)
Supplement: Figure S2 — Alignment of potential lobster IR S1 binding domains. BLAST sequence similarity searching of the olfactory transcriptome with predicted protein sequences of PargIR25a, PargIR8a, and PargIR93a, as well as from other partially sequenced lobster IRs, revealed additional potential S1 binding domains. Where possible sequences are manually aligned based on putative residue involved in ligand binding (bold/underlined). (PDF) [file pone.0060551.s002.pdf]

Supplementary Figure 2

ParargIR25a GMIKQLIDKQADIALAPLSVMAE**R**ENVVDFTVPYYDLVGITILMK
ParargIR8a GLVGDLSNGVTDLIVAPLTMTSE**R**EEVIDFVAPYFDQSGISIAMR
ParargIR93a GMVKMVSDGEVLIGVAAFSVSDQ**R**MKAVNFTTTIDRQPYAFMIAR
GMVGMVGRKESDMGVGPFSLTAV**R**AEMVDYMGLVVDDALKIIGGL
GIVGDLLDGSADLSFAPLSVTAQ**R**AKHLDFSDPYFFFSSMSILS
RPTSEWRPSTITLA**R**EEAIDFTFPYY
GMIGQVARHEAHAAICEITITYM**R**ETVVDFSYPYYLESSTLVS
GLMGQIQRHEVDIGLANLFISSH**N**LQVPDLTSPYRTE
IGLANLFNTKH**W**TQVIDLTAPYRTE
GLMGQLHRREIDIGLNAFALSIQ**W**MEVPDLTASYTAE
GLCGQLQRNEVDIGLACFYTSSH**W**LDAVELTAPYATE
QLHRNEVDIGLANLFVSNH**W**LGAADLTTPYTVE
GMVGMVGRKEVDLGLGPFGVSAT**R**AEVVDFTRSVLIDTLRIMGGV
GMVGMLLRDEAVIGLG
GMVGQVARHEAHAAFCMITISRN**R**ETVVDFTLPLL
GMVGQLERNEVDIGVANLYVSSF**Q**TEVLDFTAAYTSE
LANLYISYS**Q**KNIIEFSAPYSFE
GMVGTLTEQRADVSMMLFWSFA**R**KQVIDFTRIYT
GIIGALQHQQADFSLNIDLTST**R**IHVVDYSTLYT
GTVGVLQHQQADFSMILTLTPT**R**KHVLQYSIIYN
GMMGELQREETDFCMIAAPTPE**R**LHATEYSRGY
MVGQLQREESDLCTAVGPTP
FGLDLDLTPT**R**IHVLDYSIIYA
GIVGMLQHQQADLGLNLDLTPT**R**IHVLDYSIVYK
GTLQHQKADFSLVLSPTSG**R**DE
LNLTPA**R**TQVIEYSIVY
QRADFGLNLDLTPA**R**IHVLDYSVLY
GIIGALQHQQADFSLNIDLTST**R**IHVVDYSTLYT
GLQHQQADIGLDLDLTPK**R**IHILDYSVLYA
GTVGALQHQQADFSMLLTLTPT**R**LRVVQFSTILY
GIVGTLQHHLADFSLNLDLTPT**R**KQVLDYSILL
GIVGTLQHHKADFSLNLTPSPA**R**MKVITHTRIYS
LQHEEADFSFNLTPSPA**R**MEVITHSIIYS
GMIGMVHRKEVEFAIGPFLVTPD**R**SMVSDFSEPVYYVDNQALV
VMKEVADFATGPLSMTPA**R**GEVVDFCWPVWADNLRILGA
GMIGMVSREEVDIGVGPFTLSAT**R**AQVVDFTWPILVDHFRMLGA
TS**R**AQVVDFTGPILTEYWRVLGA
GMIGMVHRKEVEFAIGPFLVTPD**R**SMVSDFSEPVYYVDN
LGWGPFAYSAA**R**SKVIDYTRPMLIDYIRIMGG
GMVGMVGRRDADIGLGPFDVTAE**R**A
ADIGLGPLSLSAE**R**A
GMVGQVARHEAHAAFCMITISRN**R**ETVVDFTLPLL
GMLGQLQRKEAEISMGPFVMTQE**R**VTV
EIVLGPSSVSYQ**R**EAICDFSDPIVTDDFAIL
GIVGMLQHQQADLGLNLDLTPT**R**IHVLDYSIVY
EEVDIGLGPFSIRTF**R**MEMVDYTREIMFD
